# On the Acoustics of Emotion in Audio: What Speech, Music, and Sound have in Common

**DOI:** 10.3389/fpsyg.2013.00292

**Published:** 2013-05-27

**Authors:** Felix Weninger, Florian Eyben, Björn W. Schuller, Marcello Mortillaro, Klaus R. Scherer

**Affiliations:** ^1^Machine Intelligence and Signal Processing Group, Mensch-Maschine-Kommunikation, Technische Universität München, Munich, Germany; ^2^Centre Interfacultaire en Sciences Affectives, Université de Genève, Geneva, Switzerland

**Keywords:** audio signal processing, emotion recognition, feature selection, transfer learning, music perception, sound perception, speech perception

## Abstract

Without doubt, there is emotional information in almost any kind of sound received by humans every day: be it the affective state of a person transmitted by means of speech; the emotion intended by a composer while writing a musical piece, or conveyed by a musician while performing it; or the affective state connected to an acoustic event occurring in the environment, in the soundtrack of a movie, or in a radio play. In the field of affective computing, there is currently some loosely connected research concerning either of these phenomena, but a holistic computational model of affect in sound is still lacking. In turn, for tomorrow’s pervasive technical systems, including affective companions and robots, it is expected to be highly beneficial to understand the affective dimensions of “the sound that something makes,” in order to evaluate the system’s auditory environment and its own audio output. This article aims at a first step toward a holistic computational model: starting from standard acoustic feature extraction schemes in the domains of speech, music, and sound analysis, we interpret the worth of individual features across these three domains, considering four audio databases with observer annotations in the arousal and valence dimensions. In the results, we find that by selection of appropriate descriptors, cross-domain arousal, and valence regression is feasible achieving significant correlations with the observer annotations of up to 0.78 for arousal (training on sound and testing on enacted speech) and 0.60 for valence (training on enacted speech and testing on music). The high degree of cross-domain consistency in encoding the two main dimensions of affect may be attributable to the co-evolution of speech and music from multimodal affect bursts, including the integration of nature sounds for expressive effects.

## 1 Introduction

Without doubt, emotional expressivity in sound is one of the most important methods of human communication. Not only human speech, but also music and ambient sound events carry emotional information. This information is transmitted by modulation of the acoustics and decoded by the receiver – a human conversation partner, the audience of a concert, or a robot or automated dialog system. By that, the concept of emotion that we consider in this article is the one of consciously conveyed emotion (in contrast, for example, to the “true” emotion of a human related to biosignals such as heart rate). In speech, for example, a certain affective state can be transmitted through a change in vocal parameters, e.g., by adjusting fundamental frequency and loudness (Scherer et al., 2003). In music, we consider the emotion intended by the composer of a piece – and by that, the performing artist(s) as actor(s) realizing an emotional concept such as “happiness” or “sadness.” This can manifest through acoustic parameters such as tempo, dynamics (forte/piano), and instrumentation (Schuller et al., 2010). In contrast to earlier research on affect recognition from singing (e.g., Daido et al., 2011), we focus on polyphonic music – by that adding the instrumentation as a major contribution to expressivity. As a connection between music and speech emotion, for example, the effect of musical training on human emotion recognition has been highlighted in related work (Nilsonne and Sundberg, 1985; Thompson et al., 2004). Lastly, also the concept of affect in sound adopted in this article is motivated by the usage of (ambient) sounds as a method of communication – to elicit an intended emotional response in the audience of a movie, radio play, or in the users of a technical system with auditory output.

In the field of affective computing, there is currently some loosely connected research concerning either of these phenomena (Schuller et al., 2011a; Drossos et al., 2012; Yang and Chen, 2012). Despite a number of perception studies suggesting overlap in the relevant acoustic parameters (e.g., Ilie and Thompson, 2006), a holistic computational model of affect in general sound is still lacking. In turn, for tomorrow’s technical systems, including affective companions and robots, it is expected to be highly beneficial to understand the affective dimensions of “the sound that something makes,” in order to evaluate the system’s auditory environment and its own audio output.

In order to move toward such a unified framework for affect analysis, we consider feature relevance analysis and automatic regression with respect to continuous observer ratings of the main dimensions of affect, arousal, and valence, across speech, music, and ambient sound events. Thereby, on the feature side, we restrict ourselves to non-symbolic acoustic descriptors, thus eliminating more domain-specific higher-level concepts such as linguistics, chords, or key. In particular, we use a well proven set of “low-level” acoustic descriptors for paralinguistic analysis of speech (cf. Section 2.3). Then, we address the importance of acoustic descriptors for the automatic recognition of continuous arousal and valence in a “cross-domain” setting. We show that there exist large commonalities but also strong differences in the worth of individual descriptors for emotion prediction in the various domains. Finally, we carry out experiments with automatic regression on a selected set of “generic acoustic emotion descriptors.”

## 2 Materials and Methods

### 2.1 Emotion model

Let us first clarify the model of emotion employed in this article. There is a debate in the field on which type of model to adopt for emotion differentiation: discrete (categorical) or dimensional (e.g., Mortillaro et al., 2012). We believe that these approaches are highly complementary. It has been copiously shown that discrete emotions in higher dimensional space can be mapped parsimoniously into lower dimensional space. Most frequently, the two dimensions valence and arousal are chosen, although it can be shown that affective space is best structured by four dimensions – adding power and novelty to valence and arousal (Fontaine et al., 2007). Whether to choose a categorical or dimensional approach is thus dependent on the respective research context and the specific goals. Here, we chose a valence × arousal dimensional approach because of the range of affective phenomena underlying our stimuli. In addition for some of our stimulus sets only dimensional annotations were available.

### 2.2 Databases

Let us now start the technical discussion in this article by a brief introduction of the data sets used in the present study on arousal and valence in speech, music, and sound. The collection of emotional audio data for the purpose of automatic analysis has often been driven by computer engineering. This is particularly true for speech data – considering applications, for example, in human-computer interaction. This has led to large databases of spontaneous emotion expression, for example, emotion in child-robot interaction (Steidl, 2009) or communication with virtual humans (McKeown et al., 2012), which are however limited to specific domains. In contrast, there are data sets from controlled experiments, featuring, for example, emotions expressed (“enacted”) by professional actors, with restricted linguistic content (e.g., phonetically balanced pseudo sentences) with the goal to allow for domain-independent analysis of the variation of vocal parameters (Burkhardt et al., 2005; Bänziger et al., 2012). In the case of polyphonic music, data sets are mostly collected with (commercial) software applications in mind – for example, categorization of music databases on end-user devices (“music mood recognition”; Yang and Chen, 2012). Finally, emotion analysis of general sounds has been attempted only recently (Sundaram and Schleicher, 2010; Drossos et al., 2012; Schuller et al., 2012). In this light, we selected the following databases for our analysis: the Geneva Multimodal Emotion Portrayals (GEMEP) set as an example for enacted emotional speech; the Vera am Mittag (VAM) database as an example for spontaneous emotional speech “sampled” from a “real-life” context; the “Now That’s What I Call Music” (NTWICM) database for mood recognition in popular music; and the recently introduced emotional sounds database.

#### 2.2.1 Enacted emotion in speech: the Geneva multimodal emotion portrayals (GEMEP)

The GEMEP corpus is a collection of 1260 multimodal expressions of emotion enacted by 10 French-speaking actors (Bänziger et al., 2012). GEMEP comprises 18 emotions that cover all four quadrants of the arousal-valence space. The list includes the emotions most frequently used in the literature (e. g., fear, sadness, joy) as well as more subtle differentiations within emotion families (e. g., anger and irritation, fear, and anxiety). Actors expressed each emotion by using three verbal contents (two pseudo sentences and one sustained vowel) and different expression regulation strategies while they were recorded by three synchronized cameras and a separate microphone. To increase the realism and the spontaneity of the expressions, a professional director worked with the respective actor during the recording session in order to choose one scenario typical for the emotion – either by recall or mental imagery – that was personally relevant for the actor. Actors did not receive any instruction on how to express the emotion and were free to use any movement and prosody they wanted.

In the present research we consider a sub selection of 154 instances of emotional speech based on the high recognition rates reported by Bänziger et al. (2012). For this set of portrayals perceptual ratings of arousal and valence were obtained in the context of a study on the perception of multimodal emotion expressions (Mortillaro et al., unpublished). Twenty participants (10 male) listened to each of these expressions (presented in random order) and rated the content in terms of arousal and valence by using a continuous slider. Participants were given written instructions before the study. These instructions included a clear definition for each dimension that was judged. Furthermore, right before they started to rate the stimuli, they were asked whether they understood the dimensions and the two anchors and were invited to ask questions in case something was unclear. During the ratings the name of the dimension (e.g., “activation”), a brief definition (e.g., “degree of physical/physiological activation of the actor”), and the anchors (“very weak” and “very strong”) were visible on the screen.

#### 2.2.2 Spontaneous emotion in speech: the VAM corpus

The VAM corpus (Grimm et al., 2008) was collected by the institute INT of the University Karlsruhe, Germany, and consists of audio-visual recordings taken from the German TV talk show “Vera am Mittag” (English: “Vera at noon” – Vera is the name of the talk show host). In this show, the host mainly moderates discussions between guests, e.g., by occasional questions. The corpus contains 947 spontaneous, emotionally rich utterances from 47 guests of the talk show which were recorded from unscripted and authentic discussions. There were several reasons to build the database on material from a TV talk show: there is a reasonable amount of speech from the same speakers available in each session, the spontaneous discussions between talk show guests are often rather affective, and the interpersonal communication leads to a wide variety of emotional states, depending on the topics discussed. These topics were mainly personal issues, such as friendship crises, fatherhood questions, or romantic affairs. At the time of recording, all subjects did not know that the recordings were going to be analyzed in a study of affective expression. Furthermore, the selection of the speakers was based on additional factors, such as how emotional the utterances were or which spectrum of emotions was covered by the speakers, to assure a large spectrum of different and realistic affective states. Within the VAM corpus, emotion is described in terms of three basic primitives – valence, arousal, and dominance. Valence describes the intrinsic pleasantness or unpleasantness of a situation. Arousal describes whether a stimulus puts a person into a state of increased or reduced activity. Dominance is not used for the experiments reported in this article. For annotation of the speech data, the audio recordings were manually segmented to utterance level. A large number of human annotators were used for annotation (17 for one half of the data, six for the other).

For evaluation an icon-based method that consists of an array of five images for each emotion dimension was used. Each human listener had to listen to each utterance in the database to choose an icon per emotion dimension in order to best describe the emotion heard. Afterward, the choice of the icons was mapped onto a discrete five-point scale for each dimension in the range of +1 to −1, leading to an emotion estimation (Grimm et al., 2007a).

#### 2.2.3 Emotion in music: now that’s what i call music (NTWICM) database

For building the NTWICM music database the compilation “Now That’s What I Call Music!” (UK series, volumes 1–69) is selected. It contains 2648 titles – roughly a week of total play time – and covers the time span from 1983 to 2010. Likewise it represents very well most music styles which are popular today; that ranges from Pop and Rock music over Rap, R&B to electronic dance music as Techno or House. While lyrics are available for 73% of the songs, in this study we only use acoustic information.

Songs were annotated as a whole, i.e., without selection of characteristic song parts. Respecting that mood perception is generally judged as highly subjective (Hu et al., 2008), four labellers were decided for. While mood may well change within a song, as change of more and less lively passages or change from sad to a positive resolution, annotation in such detail is particularly time-intensive. Yet, it is assumed that the addressed music type – mainstream popular and by that usually commercially oriented – music to be less affected by such variation as, for example, found in longer arrangements of classical music. Details on the chosen raters are provided in Schuller et al. (2011b). They were picked to form a well-balanced set spanning from rather “naïve” assessors without instrument knowledge and professional relation to “expert” assessors including a club disc jockey (DJ). The latter can thus be expected to have a good relationship to music mood, and its perception by the audiences. Further, young raters prove a good choice, as they were very well familiar with all the songs of the chosen database. They were asked to make a forced decision according to the two dimensions in the mood plane assigning values in −2, −1, 0, 1, 2 for arousal, and valence, respectively. They were further instructed to annotate according to the perceived mood, that is, the “represented” mood, not to the induced, that is, “felt” one, which could have resulted in too high labeling ambiguity. The annotation procedure is described in detail in Schuller et al. (2010), and the annotation along with the employed annotation tool are made publicly available^1^.

#### 2.2.4 Emotion in sound events: emotional sound database

The emotional sound database (Schuller et al., 2012)^2^ is based on the on-line freely available engine FindSounds.com^3^(Rice and Bailey, 2005). It consists of 390 manually chosen sound files out of more than 10,000. To provide a set with a balanced distribution of emotional connotations, it was decided to use the following eight categories taken from FindSounds.com: *Animals*, *Musical instruments*, *Nature*, *Noisemaker*, *People*, *Sports*, *Tools*, and *Vehicles*. With this choice the database represents a broad variety of frequently occurring sounds in everyday environment. The emotional sound database was annotated by four labelers (one female, 25–28 years). They were all post graduate students working in the field of audio processing. All labelers are of Southeast-Asian origin (Chinese and Japanese), and two reported to have musical training. For the annotation these four listeners were asked to make a decision according to the two dimensions in the emotion plane assigning values on a five-point scale in {−2, −1, 0, 1, 2}for arousal and valence. They were instructed to annotate the perceived emotion and could repeatedly listen to the sounds that were presented in random order across categories. Annotation was carried out individually and independently by each of the labelers. For annotation, the procedure as described in detail in Schuller et al. (2010) was used – thus, the annotation exactly corresponds to the one used for music mood (cf. above). The annotation tool can be downloaded freely^4^.

#### 2.2.5 Reliability and “gold standard”

For all four of the databases, the individual listener annotations were averaged using the evaluator weighted estimator (EWE) as described by Grimm and Kroschel (2005). The EWE provides quasi-continuous dimensional annotations taking into account the agreement of observers. For instance *n* and dimension *d* (arousal or valence), the EWE $y_{EWE,n}^{d}$ is defined by

(1) $$y_{EWE,n}^{d}=\frac{1}{\overset{K}{\underset{k=1}{\sum }}r_{k}}\overset{K}{\underset{k=1}{\sum }}r_{k}y_{n,k}^{d},$$

where *K* is the number of labellers, and $y_{n,k}^{d}$ is the rating of instance *n* by labeller *k* in dimension *d*. Thus, the EWE is a weighted mean rating with weights corresponding to the confidence in the labeling of rater *k* – in this study, we use the correlation coefficient *r_k_* of rater *k*’s rating and the mean rating. By the first term in the above equation, the weights are normalized to sum up to one, in order to have the EWE in the same scale as the original ratings.

The average *r_k_* (across the *K* raters) is depicted for arousal and valence annotation in the four databases in Table 1. For VAM, we observe that valence was more difficult to evaluate than arousal, while conversely, on ESD, raters agree more strongly on valence than arousal. In NTWICM, both arousal and valence have similar agreement (*r* = 0.70 and 0.69). Results for GEMEP are in the same order of magnitude, indicating some ambiguity despite the fact that the emotion is enacted.

**Table 1:** **Database statistics**.

Database	Domain	Agreement [*r*]		#Annot.	#Inst.	Length [h:m]
	
		Arousal	Valence			
VAM	Speech (spontaneous)	0.81	0.56	6–17	947	0:50
GEMEP	Speech (enacted)	0.64	0.68	20	154	0:06
NTWICM	Music	0.70	0.69	4	2648	168:03
ESD	Sound	0.58	0.80	4	390	0:25

Furthermore, Table 1 summarizes the number of raters, number of rated instances, and length of the databases’ audio. It can be seen that NTWICM is by far the largest regarding the number of instances and audio length, followed by VAM, ESD, and GEMEP. The huge differences in audio length are further due to the time unit of annotation, which is similar for VAM, ESD, and GEMEP (roughly 2–4 s of audio material), yet in NTWICM entire tracks of several minutes length of popular music were rated.

**Figure 1:**
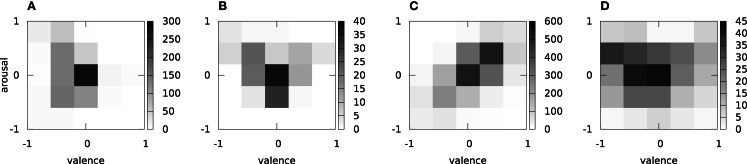
**Distribution of valence/arousal EWE on the VAM (A), GEMEP (B), emotional sound (C), and NTWICM (D) databases: number of instances per valence/arousal bin**.

Figure 1 shows the distribution of the arousal and valence EWE ratings on the three databases considered. For the purpose of this visualization, the quasi-continuous arousal/valence ratings are discretized into five equally spaced bins spanning the interval [−1, 1] on each axis, resulting in a discretization of the arousal-valence space into 25 bins. The number of instances per bin is counted. It is evident that in VAM, instances with low valence prevail – this indicates the difficulty of creating emotionally balanced data sets by sampling audio archives. Furthermore, we observe a strong concentration of ratings in the “neutral” (center) bin of the arousal-valence space. The enacted GEMEP database is overall better balanced in terms of valence and arousal ratings – yet still, there seems to be a lack of instances with low arousal and non-neutral valence rating, although some of the chosen emotion categories (e.g., pleasure) would be expected to fit in this part. For NTWICM, we observe a concentration in the first quadrant of the valence-arousal plane, and a significant correlation between the arousal and valence ratings (Spearman’s ρ = 0.61, *p* ≪ 0.001). This indicates a lack of, e.g., “dramatic” music with high arousal and low valence in the chosen set of “chart” music. Finally, in ESD, ratings are distributed all over the arousal and valence scales – as shown in more detail by Schuller et al. (2012), this is due to the different sound classes in the databases having different emotional connotation (e.g., nature sounds on average being associated with higher valence than noisemakers).

### 2.3 Extraction of acoustic descriptors

In this article, the ultimate goal is automatic emotion recognition (AER) from general sound. In contrast to neighboring fields of audio signal processing such as speech or speaker recognition, which rely exclusively on rather simple spectral cues (Young et al., 2006) as acoustic features, AER typically uses a large variety of descriptors. So far no attempt has been made at defining a “standard” feature set for generic AER from sound, which may be due to the facts that AER still a rather young field with about 15 years of active research, and that emotion recognition is a multi-faceted task owing to the manifold ways of expressing emotional cues through speech, music, and sounds, and the subjective nature of the task. Some of the currently best performing approaches for automatic speech emotion recognition (Schuller et al., 2011a) use a large set of potentially relevant acoustic features and apply a large, “brute-force” set of functionals to these in order to summarize the evolution of the contours of the acoustic features over segments of typically a few seconds in length (Ververidis and Kotropoulos, 2006). This is done to capture temporal dynamics in a feature vector of fixed length and has been shown to outperform modeling of temporal dynamics on the classifier level (Schuller et al., 2009). In the process of addressing various tasks in speech and speaker characterization in a series of research challenges (Schuller et al., 2009, 2013), various large sets for the speech domain have been proposed. Little work, however, has been done on cross-domain generalization of these features, which will be the focus of the present study.

For the analysis reported on in this article, we use a well-evolved set for automatic recognition of paralinguistic phenomena – the one of the INTERSPEECH 2013 Computational Paralinguistics Evaluation baseline (Schuller et al., 2013). In this set, suprasegmental features are obtained by applying a large set of statistical functionals to acoustic low-level descriptors (cf. Tables 2 and 3). The low-level descriptors cover a broad set of descriptors from the fields of speech processing, Music Information Retrieval, and general sound analysis. For example, Mel Frequency Cepstral Coefficients (Davis and Mermelstein, 1980; Young et al., 2006) are very frequently used in ASR and speaker identification. Further, they are used in Music Information Retrieval. Spectral statistical descriptors, such as spectral variance and spectral flux, are often used in multi-media analysis, and are part of the descriptor set proposed in the MPEG-7 multi-media content description standard (Peeters, 2004). They are thus very relevant for music and sound analysis. Loudness and energy related features are obviously important for all tasks. The same holds true for the sound quality descriptors (which are used to discriminate harmonic and noise-like sounds) and the fundamental frequency and psychoacoustic sharpness. The latter is a well-known feature in sound analysis (Zwicker and Fastl, 1999). Jitter and Shimmer are micro-prosodic variations of the length and amplitudes (respectively) of the fundamental frequency for harmonic sounds. They are mainly used in voice pathology analysis, but are also good descriptors of general sound quality.

**Table 2:** **ComParE acoustic feature set: 64 provided low-level descriptors (LLD)**.

	Group
**4 ENERGY RELATED LLD**	
Sum of auditory spectrum (loudness)	Prosodic
Sum of RASTA-style filtered auditory spectrum	Prosodic
RMS energy, zero-crossing rate	Prosodic
**55 SPECTRAL LLD**	
RASTA-style auditory spectrum, bands 1–26 (0–8 kHz)	Spectral
MFCC 1–14	Cepstral
Spectral energy 250–650 Hz, 1 k–4 kHz	Spectral
Spectral roll off point 0.25, 0.50, 0.75, 0.90	Spectral
Spectral flux, centroid, entropy, slope	Spectral
Psychoacoustic sharpness, harmonicity	Spectral
Spectral variance, skewness, kurtosis	Spectral
**6 VOICING RELATED LLD**	
*F*_0_ (SHS and viterbi smoothing)	Prosodic
Prob. of voice	Sound quality
Log. HNR, Jitter (local, delta), Shimmer (local)	Sound quality

**Table 3:** **ComParE acoustic feature set: functionals applied to LLD contours (Table 2)**.

	Group
**FUNCTIONALS APPLIED TO LLD**/**Δ** L**LD**	
Quartiles 1–3, 3 inter-quartile ranges	Percentiles
1% Percentile (≈min), 99% percentile (**≈**max)	Percentiles
Percentile range 1–99%	Percentiles
Position of min/max, range (max − min)	Temporal
Arithmetic mean^1^, root quadratic mean	Moments
Contour centroid, flatness	Temporal
Standard deviation, skewness, kurtosis	Moments
Rel. duration LLD is above 25/50/75/90% range	Temporal
Rel. duration LLD is rising	Temporal
Rel. duration LLD has positive curvature	Temporal
Gain of linear prediction (LP), LP coefficients 1–5	Modulation
Mean, max, min, SD of segment length^2^	Temporal
**FUNCTIONALS APPLIED TO LLD ONLY**	
Mean value of peaks	Peaks
Mean value of peaks – arithmetic mean	Peaks
Mean/SD of inter peak distances	Peaks
Amplitude mean of peaks, of minima	Peaks
Amplitude range of peaks	Peaks
Mean/SD of rising/falling slopes	Peaks
Linear regression slope, offset, quadratic error	Regression
Quadratic regression a, b, offset, quadratic error	Regression
Percentage of non-zero frames^3^	Temporal

^1^Arithmetic mean of LLD/positive Δ LLD. ^2^Not applied to voice related LLD except *F*_0_. ^3^Only applied to F0.

## 3 Results

### 3.1 Feature relevance

Let us now discuss the most effective acoustic features out of the above mentioned large set for single- and cross-domain emotion recognition. To this end, besides correlation coefficients (*r*) of features with the arousal or valence ratings, we introduce the cross-domain correlation coefficient (CDCC) as criterion. As we strive to identify features which carry similar meaning with respect to emotion in different domains, and at the same time provide high correlation with emotion in the domains by themselves, the purpose of the CDCC measure is to weigh high correlation in single domains against correlation deviations across different domains. Let us first consider a definition for two domains *i* and *j*, namely

(2) $${\text{CDCC}}_{f,i,j}^{2}=\frac{\left|r_{f}^{\left(i\right)}+r_{f}^{\left(j\right)}\right|-\left|r_{f}^{\left(i\right)}-r_{f}^{\left(j\right)}\right|}{2}$$

where $r_{f}^{\left(i\right)}$ is the correlation of feature *f* with the domain *i*, and “domain” refers to the arousal or valence annotation of a certain data set. We only consider the CDCC across the data sets (speech, music, and sound), not CDCC across arousal and valence.

It is obvious that the CDCC measure is symmetric in the sense that ${\text{CDCC}}_{f,i,j}^{2}={\text{CDCC}}_{f,j,i}^{2}$, and that it ranges from −1 to 1. If a feature f exhibits either strong positive or strong negative correlation with both domains, the CDCC will be near one, where as it will be near −1 if a feature is strongly positively correlated with one domain yet strongly negatively correlated with the other. A CDCC near zero indicates that the feature is not significantly correlated with both domains (although it might still be correlated with either one). Thus, we can expect a regressor to show similar performance on both domains if it uses features with high CDCC.

Next, we generalize the CDCC^2^ to *J* domains by summing up the CDCCs for domain pairs and normalizing to the range from −1 to +1,

(3) $${\text{CDCC}}_{f}^{J}=\frac{\sum_{i=1}^{J}\sum_{j=i+1}^{J}\left(\left|r_{f}^{\left(i\right)}+r_{f}^{\left(j\right)}\right|-\left|r_{f}^{\left(i\right)}-r_{f}^{\left(i\right)}\right|\right)}{J\left(J-1\right)}.$$

Intuitively, a regression function determined on features with high ${\text{CDCC}}_{f}^{J}$ is expected to generalize well to all *J* domains.

In Tables 4 and 5, we now exemplify the CDCC^3^ across the three domains on selected features, along with presenting their correlation on the individual domains. Note that for the purpose of feature selection, we treat the union of VAM and GEMEP as a single domain (“speech”). Further, in our analysis we restrict ourselves to those features that exhibit high (absolute) correlation in a single domain (termed *sound, speech*, or *music features* in the table), and those with high CDCC^3^ (termed *cross-domain features*). Thereby we do not present an exhaustive list of the top features but rather a selection aiming at broad coverage of feature types. To test the significance of the correlations, we use t-tests with the null hypothesis that feature and rating are sampled from independent normal distributions. Two-sided tests are used since we are interested in discovering both negative and positive correlations. Significance levels are adjusted by Bonferroni correction, which is conservative, yet straightforward and does not require independence of the individual error probabilities.

**Table 4:** **Cross-domain feature relevance for arousal: top features ranked by absolute correlation (*r*) for single domain, and CDCC across all three domains (CDCC^3^)**.

Rank	LLD	Functional	*r*			CDCC^3^
	
			Sound	Music	Speech
**SOUND FEATURES**						
1	Loudness	R.q. mean	0.59**	0.16**	0.75**	0.31
4	Loudness	Lin. regr. offset	0.54**	0.27**	0.56**	0.36
6	Loudness	99-Percentile	0.53**	0.09 °	0.67**	0.23
8	Energy	R.q. mean	0.50**	0.07^−^	0.64**	0.21
**SPEECH FEATURES**						
1	Spectral flux	R.q. mean	0.38**	0.13**	0.76**	0.21
9	Δ Spectral flux	Arith. mean	0.25*	0.28**	0.68**	0.26
63	Δ MFCC 14	R.q. mean	0.14^−^	0.32**	0.58**	0.20
97	F0	R.q. mean	0.17^−^	0.09^o^	0.55**	0.12
**MUSIC FEATURES**						
1	Loudness	Mean peak dist.	0.02^−^	–0.58**	–0.08^−^	0.01
2	Spectral ent.	Mean peak dist.	0.04^−^	–0.54**	–0.16**	0.03
3	Loudness	Peak dist. SD	0.02^−^	–0.53**	–0.10^−^	0.02
5	MFCC 1	R.q. mean	–0.11^−^	–0.53**	–0.47**	0.23
**CROSS-DOMAIN FEATURES**						
1	Loudness	Quad.reg. offset	0.41**	0.37**	0.37**	0.37
4	Loudness	Arith. mean	0.57**	0.18**	0.73**	0.31
5	Spectral flux	Quad. reg. offset	0.32**	0.30**	0.45**	0.31
6	Δ Energy 1–4 kHz	Quartile 1	–0.32**	–0.30**	–0.59**	0.31

Significance denoted by **p < 0.00, *p < 0.01, °*p* < 0.05, ^−^*p* ≥ 0.05; Bonferroni corrected *p*-values from two-sided paired sample t-tests.

**Table 5:** **Cross-domain feature relevance for valence: top features ranked by absolute correlation (*r*) for single domain, and CDCC across all three domains (CDCC^3^)**.

Rank	LLD	Functional	*r*			CDCC^3^
	
			Sound	Music	Speech
**SOUND FEATURES**						
1	Loudness	Quartile 3	–0.31**	0.27**	–0.21**	–0.09
2	Loudness	Rise time	–0.30**	–0.21**	–0.04^−^	0.10
3	Loudness	R.q. mean	–0.29**	0.29**	–0.23**	–0.10
10	Spectral flux	Skewness	0.27**	–0.13**	0.11^−^	–0.04
**SPEECH FEATURES**						
1	F0	Quartile 2	0.05^−^	–0.07^−^	–0.31**	–0.01
2	Energy 1–4 kHz	Arith. mean	–0.17^−^	0.23**	–0.31**	–0.07
4	Δ Energy 1–4 kHz	Arith. mean	–0.08^−^	0.26**	–0.30**	–0.09
10	F0	Quartile 1	0.07^−^	–0.14**	–0.29**	0.00
**MUSIC FEATURES**						
1	Δ Loudness	Mean peak dist.	–0.02^−^	–0.65**	–0.03^−^	0.02
2	Loudness	Mean peak dist.	–0.12^−^	–0.65**	–0.04^−^	0.06
3	MFCC 1	Quartile 2	–0.04^−^	–0.61**	0.24**	–0.08
9	Spectral ent.	Mean peak dist.	0.05^−^	–0.57**	0.04^−^	–0.02
**CROSS-DOMAIN FEATURES**						
1	Spect. centroid	Rise time	–0.13^−^	–0.16**	–0.12^−^	0.12
2	Psy. sharpness	Rise time	–0.13^−^	–0.16**	–0.12^−^	0.12
5	Energy 250–650 Hz	IQR 1–3	–0.14^−^	–0.11**	–0.15*	0.12
6	MFCC 13	IQR 1–3	–0.08^−^	–0.20**	–0.18**	0.12

Significance denoted by **p < 0.001, *p < 0.01, ^o^*p* < 0.05), ^−^*p* ≥ 0.05; Bonferroni corrected *p*-values from two-sided paired sample t-tests.

Looking at the top sound arousal features (Table 4), we find loudness to be most relevant – in particular, the (root quadratic) mean, the linear regression offset (corresponding to a “floor value”) and the 99-percentile. This is similar to the ranking for speech. Interestingly, loudness is stronger correlated than RMS energy, indicating the importance of perceptual auditory frequency weighting as performed in our loudness calculation. For music, these three loudness features are not as relevant, though still significantly correlated.

The overall best speech arousal feature is the root quadratic mean of spectral flux – indicating large differences of consecutive short-time spectra – which is interesting since it is independent of loudness and energy, which have slightly lower correlation (cf. above). The “second derivative” of the short-time spectra (arithmetic mean of Δ spectral flux) behaves in a similar fashion as spectral flux itself. However, the correlation of these features with arousal in sound and music is lower. Further, we find changes in the higher order MFCCs, such as the root quadratic mean of delta MFCC 14 to be relevant for speech and music arousal, relating to quick changes in phonetic content and timbre. Finally, mean F0, a “typical” speech feature characteristic for high arousal, is found to be relevant as expected, but does not generalize to the other domains.

The best music arousal features are related to mean peak distances – for example, in the loudness contour and the spectral entropy contour resembling occurrence of percussive instruments, indicating positive correlation between tempo and arousal. In contrast, the peak distance standard deviation is negatively correlated with arousal – thus, it seems that “periodic” pieces of music are more aroused, which can be explained by examples such as dance music. However, it seems that all these three features have a mostly musical meaning, since they only show weak correlations in sound and speech. Yet, a notable feature uniting speech and music is the (root quadratic) mean of the first MFCC, which is related to spectral skewness: arguably, a bias toward lower frequencies (high skewness) is indicative of absence of broadband (mostly percussive) instruments, and “calm” voices, and thus low arousal.

Summarizing cross-domain features for arousal, we find that the “greatest common divisor” of speech, sound, and music is loudness (and – relatedly – energy), but the behavior of functional types is interesting: the quadratic regression offset is much more relevant in the case of music than the mean loudness, which is mostly characteristic in speech and sound. In the NTWICM database of popular music, in fact we often find parabola shaped loudness contours, such that this offset indicates the intensity of the musical climax. A suitable cross-domain feature not directly related to loudness or energy is the spectral flux quadratic regression offset (the ordinate of the “high point” of spectral change).

Judging from the results in Table 5, we see that loudness is also indicative of *valence* in sound, music, and speech, but the correlations have different signs: on the one hand, loud sounds as identified by high root quadratic mean of loudness are apparently perceived as unpleasant, as are loud voices. For music, on the other hand, loudness can be indicative of high valence (“happy” music).

Among relevant speech valence features, we find mean energy (change) in the speech frequency range (1–4 kHz) and F0 (quartiles 1 and 2) – F0, however, is a “speech only” feature which exhibits low correlation in the other domains (similarly to the observations for arousal above).

Music valence features overlap with music arousal features, due to the correlation in the ratings. Among the music valence features, the median first MFCC (related to spectral skewness – cf. above) is particularly noticeable as it has “inverse” correlation on speech and music – “percussive” music with a flat spectrum is connotated with positive emotion (high valence) while “noisy” voices are characteristic of negative emotion (low valence).

Cross-domain features for valence are generally rarely significant on the individual domains and hard to interpret – here, in contrast to arousal, it seems difficult to obtain descriptors that generalize across multiple domains.

We now move from discussion of single features to a broader perspective on automatic feature selection for cross-domain emotion recognition. To this end, we consider automatically selected subsets of the ComParE feature set by the CDCC criteria. In particular, for each of arousal and valence, we choose the 200 features that show the highest CDCC^2^ for the (sound, music), (sound, speech), and (music, speech) pairs of domains. Furthermore, for each of arousal and valence, we select a set of 200 features by highest CDCC^3^ across all three of the sound, music, and speech domains.

**Figure 2:**
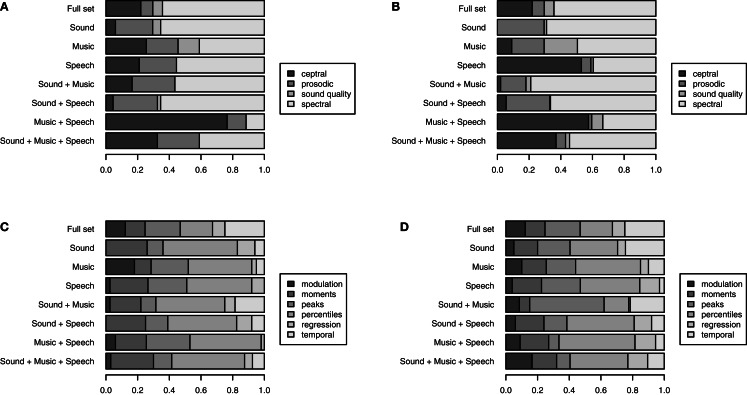
**Feature relevance by LLD group (A: arousal, B: valence) and functional group (C: arousal, D: valence)**. Number of features in the top 200 features ranked by absolute correlation with gold standard for single domain and CDCC [equation (3)] for cross domain.

In Figure 2, we summarize the obtained feature sets by the share of cepstral, prosodic, spectral, and voice quality LLDs, as well as by the share of modulation, moment, peak, percentile, regression, and temporal functionals (see Tables 2 and 3 for a list of descriptors in each of these groups). We compare the cross-domain feature sets to the full ComParE feature set as well as the “single domain” feature sets that are obtained in analogy to the cross-domain feature sets by applying the CDCC^2^ to a 50% split of each corpus. A feature group is considered particularly relevant for a recognition task if its share among the selected features is larger than its share of the full feature set.

We observe notable differences in the importance of different LLD groups; it is of particular interest for the present study to highlight the results for the considered cross-domain emotion recognition tasks: cepstral features seem to be particularly relevant for cross-domain speech and music emotion recognition. In contrast, cross-domain emotion recognition from speech and sound, and from sound and music, are dominated by “prosodic” and spectral cues such as loudness, sub-band energies, and spectral flux. Regarding relevant functional types, the summarization reveals less evident differences between the tasks; still, percentile type functionals seem to be particularly promising for all of the tasks considered.

### 3.2 Automatic classification experiments

Finally, we demonstrate the predictive power of the obtained cross-domain feature sets in automatic regression. In automatic regression, the parameters of a regression function on N-dimensional feature vectors are optimized to model the assignment of L “learning” vectors (e.g., feature vectors of emotional utterances) to the gold standard (e.g., the arousal observer rating). Then, the regression function is evaluated on a disjoint set of test vectors and the correlation of the function’s predictions and the test set gold standard is computed as a measure of how well the regression function generalizes to “unseen” test data. In the present study, it is of particular interest to consider cross-domain evaluation, i.e., training on data from one domain (e.g., enacted speech) and evaluating on another domain (e.g., sound). In this context, we also treat spontaneous and enacted speech as different domains, as such analysis is receiving increasing attention at the moment (Bone et al., 2012) also due to practical reasons: for instance, it is of interest to determine if training on “prototypical” data from a controlled experiment (such as the GEMEP database) can improve automatic emotion recognizers applied “in the wild,” e.g., to media analysis (such as given by the VAM database). For reference, we also consider within-domain regression in a twofold cross-validation manner.

For each learning set, we determine a multivariate linear regression function by means of support vector regression (SVR) (Smola and Schölkopf, 2004), which defines a real valued mapping

(4) $$f\left(\text{x}\right)={\text{w}}^{T}\text{x}+b$$

of *N*-dimensional feature vectors **x** to a regression value *f* (**x**). **w** is the normal vector of the *N*-dimensional hyperplane describing the regression function, and *b* is a scalar offset. Specifically for SVR, the primary optimization goal is *flatness* of the regression function, which is defined as low norm of the weight vector **w**. This is related to the notion of *sparsity* and crucial to avoid over-fitting of the model parameters in the present case of high dimensional feature spaces. The trade-off between flatness of the weight vector and deviation of the regression values from the gold standard on the learning set is modeled as a free parameter *C* in the optimization (cf. Smola and Schölkopf, 2004 for details). In our experiments, *C* is set to 10^−5^ for within-domain regression and 10^−5^ for cross-domain regression. The optimization problem is solved by the frequently used Sequential Minimal Optimization algorithm (Platt, 1999). To foster reproducibility of our research, we use the open-source machine learning toolkit Weka (Hall et al., 2009). Unsupervised mean and variance normalization of each feature per database is applied since SVR is sensitive to feature scaling.

In Table 6, the correlation coefficients (*r*) of automatic within-domain and cross-domain regression with the arousal observer ratings are displayed. First, we consider regression using the full 6373-dimensional ComParE feature set. In within-domain regression, results ranging from *r* = 0.54 (sound) up to *r* = 0.85 (enacted speech) are obtained, which are comparable to previously obtained results on sound, music, and spontaneous speech (Grimm et al., 2007b; Schuller et al., 2011b, 2012). Especially the result for music is notable, since we do not use any “hand-crafted” music features such as chords or tempo. In cross-domain regression, significant correlations are obtained except for the case of training on music and evaluating on sound. However, the mean r across all training and testing conditions (0.50) is rather low.

**Table 6:** **Results of within-domain and pair-wise cross-domain support vector regression on arousal observer ratings for sound (emotional sound database), music (NTWICM database), and spontaneous and enacted speech (VAM/GEMEP databases)**.

*r*	Test on				Mean
	
Train on	Sound	Music	Speech	
	
			Sp.	En.
**(A) FULL FEATURE SET**					
Sound	0.54**	0.14**	0.70**	0.64**	0.51
Music	0.11^−^	0.65**	0.46**	0.39**	0.40
Speech/Sp.	0.38**	0.37**	0.81**	0.80**	0.59
Speech/En.	0.20*	0.32**	0.60**	0.85**	0.49
Mean	0.30	0.37	0.64	0.67	0.50
**(B) 200 TASK-SPECIFIC FEATURES**					
Sound	0.59**	0.46**	0.76**	0.79**	0.65
Music	0.46**	0.67**	0.73**	0.75**	0.65
Speech/Sp.	0.54**	0.47**	0.83**	0.78**	0.66
Speech/En.	0.56**	0.46**	0.77**	0.85**	0.66
Mean	0.54	0.52	0.77	0.79	0.65
**(C) 200 GENERIC FEATURES**					
Sound	0.56**	0.35**	0.78**	0.56**	0.56
Music	0.38**	0.66**	0.74**	0.63**	0.60
Speech/Sp.	0.44**	0.43**	0.82**	0.69**	0.59
Speech/En.	0.31**	0.45**	0.77**	0.78**	0.58
Mean	0.42	0.47	0.78	0.67	0.58

Significance denoted by **p < 0.001, *p < 0.01, ^−^*p* ≥ 0.05; Bonferroni corrected *p*-values from two-sided paired sample t-tests. Full ComParE feature set (cf. Tables 2 and 3); 200 top features selected by CDCC^2^ for specific within-domain or cross-domain regression tasks; Generic features: 200 features selected by CDCC^3^ across sound, music, and speech domains (cf. Table 4).

Considering automatic feature selection by CDCC^2^ for each combination of two domains, results in Table 6B indicate a drastic gain in performance especially for cross-domain regression. However, also the results in within-domain regression are improved. All correlations are significant at the 0.1% level. Particularly, using CDCC based feature selection robust regression (achieving *r* > 0.76) is possible across enacted and spontaneous speech. Further, it is notable that the average result across the four testing databases does not vary much depending on the training database used, indicating good generalization capability of the selected features. The overall mean r in this scenario is 0.65.

Finally, if we select the top features by CDCC^3^ on all databases (treating speech as a single domain for the purpose of feature selection), it is notable that we still obtain reasonable results (mean r of 0.58) despite the fact that the top features by CDCC^3^ exhibit comparably low correlation with the target labels on the single domains (cf. Table 4).

Summarizing the results for valence regression (Table 7), we observe that using the full feature set, we cannot obtain reasonable results in cross-domain regression. Among the cross-domain results, the only significant positive correlations are obtained in evaluation on spontaneous speech, however, these are lower than the correlation of the single best speech features. Interestingly, we observe significant negative correlations when evaluating on music and training on another domain, which is consistent with the fact that some of the music valence features are “inversely” correlated with the target label in the other domains (cf., e.g., the discussion of median MFCC 1 above). In the within-domain setting, it can be observed that regression on valence in music is possible with high robustness (*r* = 0.80). This is all the more noticeable since this correlation is higher than the one obtained in arousal regression, while for the other domains, valence seems to be harder to recognize than arousal. This can partly be attributed to the fact that in the analyzed music data, the valence rating is correlated to the arousal rating.

**Table 7:** **Results of within-domain and pair-wise cross-domain support vector regression on valence observer ratings for sound (emotional sound database), music (NTWICM database), and spontaneous and enacted speech (VAM/GEMEP databases)**.

*r*	Test on				Mean
	
Train on	Sound	Music	Speech	
	
			Sp.	En.
**(A) FULL FEATURE SET**					
Sound	0.40**	−0.11**	0.21**	−0.02^−^	0.12
Music	−0.17 °	0.80**	−0.13*	0.08^−^	0.15
Speech/Sp.	0.11^−^	−0.15**	0.46**	0.21^−^	0.16
Speech/En.	−0.06^−^	−0.18**	0.12*	0.26^o^	0.03
Mean	0.07	0.09	0.17	0.13	0.12
**(B) 200 TASK-SPECIFIC FEATURES**					
Sound	0.51**	0.36**	0.27**	0.48**	0.41
Music	0.40**	0.82**	0.33**	0.52**	0.52
Speech/Sp.	0.30**	0.45**	0.44**	0.26^o^	0.36
Speech/En.	0.45**	0.60**	0.36**	0.50**	0.48
Mean	0.41	0.56	0.35	0.44	0.44
**(C) 200 GENERIC FEATURES**					
Sound	0.26**	0.41**	0.27**	0.12^−^	0.27
Music	0.27**	0.75**	0.33**	0.25^o^	0.40
Speech/Sp.	0.20*	0.45**	0.35**	0.19^−^	0.30
Speech/En.	0.20**	0.44**	0.32**	0.23^−^	0.30
Mean	0.23	0.52	0.32	0.20	0.32

Significance denoted by **p < 0.001, *p < 0.01, °*p* < 0.05, ^−^*p* ≥ 0.05; Bonferroni corrected *p*-values from two-sided paired sample t-tests. Full ComParE feature set (cf. Tables 2 and 3); 200 top features selected by CDCC^2^ for specific within-domain or cross-domain regression tasks; Generic features: 200 features selected by CDCC^3^ across sound, music, and speech domains (cf. Table 5).

Concerning feature selection by CDCC^2^ (Table 7B), we observe a boost in the obtained correlations (mean = 0.44, compared to 0.12 without feature selection). For instance, when training on enacted speech and evaluating on music, we obtain a significant *r* of 0.60. This result is interesting in so far as the best selected feature for this particular cross-domain setting, namely the flatness of the loudness contour, only exhibits a correlation of 0.28, respectively 0.27, with the valence rating on the NTWICM (music) and GEMEP (enacted speech) databases. Thus, the 200 CDCC^2^-selected features for this regression task seem to be of complementary nature. Furthermore, by applying feature selection in the within-domain setting, best results are obtained for sound (*r* = 0.51), music (*r* = 0.82), and enacted speech (*r* = 0.50) valence recognition. However, regarding the issue of enacted vs. spontaneous speech, we find that regressors trained on one type do not generalize well to the other, which is in contrast to the finding for arousal.

Finally, when applying the “generic valence feature set” obtained from the CDCC^3^ ranking across sound, music, and speech, we obtain an average correlation of 0.32. Results are considerably below the CDCC^2^ results particularly for sound and enacted speech. This – again – points at the difficulty of finding features that generalize to valence recognition across domains. However, it is notable that robust results (*r* = 0.75) are obtained in within-domain music recognition using the generic feature set, of which the “best” feature (rise time of spectral centroid) only has an (absolute) correlation of 0.16 with the music valence rating.

## 4 Discussion

We have presented a set of acoustic descriptors for emotion recognition from audio in three major domains: speech (enacted and spontaneous), music, and general sound events. Using these features, we have obtained notable performances in within-domain regression – particularly, these surpass the so far best published results on the NTWICM database (Schuller et al., 2011b) despite the fact that the latter study used hand-crafted music features rather than the generic approach pursued in the present paper.

We have found that it is rather hard to obtain features that are equally well correlated across the three domains. For arousal, such features comprise mostly loudness-related ones. In contrast, we have not been able to obtain features that are significantly correlated with the valence rating in all domains. A further notable result for valence is that some features have an “inverse” meaning in different domains (i.e., significant correlations with different signum), while this does not occur for arousal. It will be subject of further research whether this is simply due to the correlation of intended arousal and valence in popular music or to more fundamental differences.

This phenomenon has motivated the introduction of a “cross-domain correlation coefficient” which summarizes the differences in correlation across multiple domains. Using this coefficient, we were able to provide an automatic method of selecting generalizing features for cross-domain arousal and valence recognition. In the result, cross-domain arousal and valence regression has been proven feasible, achieving significant correlations with the observer annotations.

The degree of cross-domain consistency in encoding the two main dimensions of affect – valence and arousal – demonstrated in this article is quite astounding. Music has often been referred to as the “language of emotion” and a comprehensive review of empirical studies on the expression of emotion in speech and music (Juslin and Laukka, 2003) has confirmed the hypothesis that the acoustic parameters marking certain emotions are quite similar in music and speech (cf. also Ilie and Thompson, 2006). Scherer (1991) has suggested that speech and emotion may have evolved on the basis of primitive affect bursts serving similar communicative functions across many mammalian species. Ethological work shows that expression and impression are closely linked, suggesting that, in the process of conventionalization and ritualization, expressive signals may have been shaped by the constraints of transmission characteristics, limitations of sensory organs, or other factors. The resulting flexibility of the communication code is likely to have fostered the evolution of more abstract, symbolic language, and music systems, in close conjunction with the evolution of the brain to serve the needs of social bonding and efficient group communication.

As vocalization, which remained a major modality for analog emotion expression, became the production system for the highly formalized, segmental systems of language and singing, both of these functions needed to be served at the same time. Thus, in speech, changes in fundamental frequency (F0), formant structure, or characteristics of the glottal source spectrum can, depending on the language and the context, serve to communicate phonological contrasts, syntactic choices, pragmatic meaning, or emotional expression. Similarly, in music, melody, harmonic structure, or timing may reflect the composer’s intentions, depending on specific traditions of music, and may simultaneously induce strong emotional moods. This fusion of two signal systems, which are quite different in function and in structure, into a single underlying production mechanism, vocalization, has proven to be singularly efficient for the purpose of communication, and the relatively high degree of convergence as demonstrated by the correlations found in our study suggests that it might be possible to identify elements of a common code for emotion signaling. Recently, Scherer (2013) has reviewed theoretical proposals and empirical evidence in the literature that help to establish the plausibility of this claim, in particular, the evolutionary continuity of affect vocalizations, showing that anatomical structures for complex vocalizations existed before the evidence for the presence of representational systems such as language.

As to the cross-domain consistency with different kinds of environmental sounds, it seems quite plausible to assume that once speech and music were decoupled from actually occurring affect bursts and took on representational functions, different kinds of nature sounds were used in speech and music both for reference to external events and expressive functions. It seems reasonable to assume that the type of representational coding was informed by the prior, psychobiological affect code, particularly with respect to the fundamental affect dimensions of valence and arousal.

Empirical studies like the one reported here, using machine learning approaches, may complement other approaches to examine the evolutionary history of affect expression in speech and music by empirically examining, using large corpora of different kinds of sound events, the extent to which auditory domains exhibit cross-domain consistency and which common patterns are particularly frequent.

## Conflict of Interest Statement

The authors declare that the research was conducted in the absence of any commercial or financial relationships that could be construed as a potential conflict of interest.

## Footnotes

^1^http://openaudio.eu/NTWICM-Mood-Annotation.arff – accessed 27 Mar 2013

^2^http://www.openaudio.eu/Emotional-Sound-Database.csv – accessed 27 Mar 2013

^3^http://www.findsounds.com – accessed 27 Mar 2013

^4^http://www.openaudio.eu/wsh_mood_annotation.zip – accessed 27 Mar 2013
